# Vertical variation in leaf functional traits of *Parashorea chinensis* with different canopy layers

**DOI:** 10.3389/fpls.2024.1335524

**Published:** 2024-01-29

**Authors:** Nan Jin, Xiaocheng Yu, Jinlong Dong, Mengcheng Duan, Yuxuan Mo, Leiyun Feng, Rong Bai, Jianli Zhao, Jia Song, Gbadamassi Gouvide Olawole Dossa, Huazheng Lu

**Affiliations:** ^1^ School of Ecology and Environment Science, Yunnan University, Kunming, China; ^2^ CAS Key Laboratory of Tropical Forest Ecology, Xishuangbanna Tropical Botanical Garden, Chinese Academy of Sciences, Mengla, China; ^3^ National Forest Ecosystem Research Station at Xishuangbanna, Xishuangbanna Tropical Botanical Garden, Chinese Academy of Sciences, Mengla, China; ^4^ Xishuangbanna Forest Ecosystem Yunnan Field Scientific Observation Research Station, Xishuangbanna Tropical Botanical Garden, Chinese Academy of Sciences, Xishuangbanna Tropical Botanical Garden, Chinese Academy of Sciences, Mengla, China; ^5^ Qianyanzhou Ecological Research Station, Key Laboratory of Ecosystem Network Observation and Modeling, Institute of Geographic Sciences and Natural Resources Research, Chinese Academy of Sciences, Beijing, China; ^6^ School of Environmental and Geographical Science, Shanghai Normal University, Shanghai, China

**Keywords:** canopy physiology, intraspecific variation, leaf hydraulic traits, leaf photosynthetic physiological traits, vertical distribution, ecological adaptation

## Abstract

**Introduction:**

Canopy species need to shift their ecological adaptation to improve light and water resources utilization, and the study of intraspecific variations in plant leaf functional traits based at individual scale is of great significance for evaluating plant adaptability to climate change.

**Methods:**

In this study, we evaluate how leaf functional traits of giant trees relate to spatial niche specialization along a vertical gradient. We sampled the tropical flagship species of *Parashorea chinensis* around 60 meters tall and divided their crowns into three vertical layers. Fourteen key leaf functional traits including leaf morphology, photosynthetic, hydraulic and chemical physiology were measured at each canopy layer to investigate the intraspecific variation of leaf traits and the interrelationships between different functional traits. Additionally, due to the potential impact of different measurement methods (*in-situ* and *ex-situ* branch) on photosynthetic physiological parameters, we also compared the effects of these two gas exchange measurements.

**Results and discussion:**

*In-situ* measurements revealed that most leaf functional traits of individual-to-individual *P. chinensis* varied significantly at different canopy heights. Leaf hydraulic traits such as midday leaf water potential (MWP) and leaf osmotic potential (OP) were insignificantly correlated with leaf photosynthetic physiological traits such as maximal net assimilation rate per mass (*A*_mass_). In addition, great discrepancies were found between *in-situ* and *ex-situ* measurements of photosynthetic parameters. The *ex-situ* measurements caused a decrease by 53.63%, 27.86%, and 38.05% in *A*_mass_, and a decrease of 50.00%, 19.21%, and 27.90% in light saturation point compared to the *in-situ* measurements. These findings provided insights into our understanding of the response mechanisms of *P. chinensis* to micro-habitat in Xishuangbanna tropical seasonal rainforests and the fine scale adaption of different resultant of decoupled traits, which have implications for understanding ecological adaption strategies of *P. chinensis* under environmental changes.

## Introduction

Forests are highly diversified ecosystems that play a key role in the carbon and hydrologic cycle (Needham et al., 2022). Some far anthropogenic influences on global climate change have led to longer and more frequent climate extremes, which may adversely affect the structure and function of forest communities. In response, plants adjust physiologically and morphologically to changes in their surrounding climate, thereby forming specific functional traits, reflecting adaptation strategies to different environment stresses. Consequently, trait-based ecological research has potential to account for plant species distribution and function, perfect global vegetation patterns, and to predict ecosystem processes (Wright et al., 2004; Díaz et al., 2016; Shi et al., 2020; Hagan et al., 2023). However, prior studies mainly consider the trait characteristics by hypothesizing that the individuals of plant species are the same (Anderegg et al., 2018). This approach overlooks the fact that intraspecific variations in plant functional traits across individuals can contribute significantly to the total trait variation of a plant population even a forest community (Huang et al., 2023). Intraspecific variations represent plant genetic diversity and the different phenotypes produced by the same genotype under variable ambient conditions, which is well known as phenotypic plasticity (Sobral and Sampedro, 2022). The overlook in the intraspecific variation may lead to inaccurate estimation on trait diversity (Lasky et al., 2014; Siefert et al., 2015). In comparison with the patterns observed at the species level, the diversity of individual traits may provide more compelling evidence for ecological adaptation and indicate the different axes of niche division (Koch et al., 2004; Lasky et al., 2014; Pfautsch et al., 2018).

Prior studies focusing on functional trait variations in leaves have greatly advanced our comprehension of plant performance and ecological adaptation. Among various leaf-level traits, those related to CO_2_ and water exchange and light capture have probably received more attention (Li et al., 2015; Xu et al., 2021). Leaf economics and hydraulic traits directly affect plant nutrient absorption and carbon assimilation rate, and control water transport to resist atmospheric drought (Wright et al., 2004). This in turn sets limitations on key aspects of plant architecture such as maximum height, allometry and canopy physiology (Koch et al., 2004; de Bittencourt et al., 2022). Forest microenvironment is an important driver of the intraspecific variation of leaf functional traits (Kenzo et al., 2015; Zuleta et al., 2022). Forest ecosystems experience notable environmental heterogeneity along vertical gradients, with increasing light radiation and vapor pressure difference (VPD) and decreasing atmospheric humidity (Duan et al., 2022). Within the same forest community, changes in local environmental gradients, which often follow the same patterns as those found at large spatial scales, can strongly influence ecological adaptation strategies (Kenzo et al., 2015; Lu et al., 2015; Chen et al., 2019). Moreover, leaf also play a pivotal role in niche differentiation (Ishii et al., 2013; Hess et al., 2022). In tropical and subtropical regions, ecological adaptation along a fine-scale vertical gradient can be explained by habitat filtering based on leaf traits (Kenzo et al., 2015; Feng et al., 2022). Leaves in these regions are highly specialised to the microenvironment conditions, exhibiting contrasting physiological characteristics found under strong selective pressures (Koch et al., 2004). Thus, traits related to how efficiently plants carbon assimilation and resist drought stress are highlighted as critical factors on the mechanism of species coexistence in a community (Li et al., 2015).

Given that leaf economics and hydraulic traits are both related to the process of water-CO_2_ exchange, the relationship of these two sets of traits has garnered widespread attention (Li et al., 2015). From the perspective of a single economics spectrum, there is a strong coordination between leaf economics and hydraulic traits across comprehensive species, implying that leaves with higher photosynthetic rates tend to have more effective hydraulic systems (Wright et al., 2004; Reich, 2014). Nevertheless, leaf functional traits may not always change in this coordinated way. Increasing evidence supports multiple dimensions of plant functional traits (Sack et al., 2003; Sack and Scoffoni, 2013). Recent studies in tropical-subtropical forest systems suggested that there was a decoupling between leaf hydraulic and economics traits among different species (Li et al., 2015). Blackman et al. (2016) further found that leaf functional trait coordination at intraspecific scale was also decoupled in response to environmental change. This decoupling helps for understanding the ecological adaptation strategies of species under climate change, because these independent trait dimensions may be selected by different environmental filters (Li et al., 2015). Despite this, whether this phenomenon also exists at the individual scale remains to be discussed, as there is little research on the relationship between different types of functional traits in specific species (Wright et al., 2004). In complex forest ecosystems where resource space is multidimensional and highly heterogeneous, to improve the performance of whole tree performance, different combinations of leaf functional traits at the individual species level is likely to be necessary to adapt to various changes in fine-scale habitats (Martinez and Fridley, 2018; Pritzkow et al., 2020).

*Parashorea chinensis* H. Wang, belonging to the family Dipterocarpaceae, is the flagship species in the Asian tropics (Tang et al., 2008; de Bittencourt et al., 2022). *P. chinensis* is distributed in the tropical seasonal rainforest of China, especially in Xishuangbanna, Yunnan Province. Adult individuals of *P. chinensis* in the canopy may reach heights above 60 m (Deng et al., 2020), which is also the tallest tree of tropical seasonal rainforest in China and means a high proportion of aboveground carbon storage (Tang et al., 2008). Meanwhile, *P. chinensis* also plays an essential role in maintaining biodiversity and the stability of ecological networks in community (Hu et al., 2022; Shen et al., 2022). Data reveal that large trees are more susceptible to the impact of drought due to changes in precipitation time and amplitude, as well as increasing temperatures (Bennett et al., 2015; Liu et al., 2021). Given that the structure of these forests is highly variable across the vertical gradient (Deng et al., 2020; Shen et al., 2022), if the adult *P. chinensis* fails to respond adaptively to keep up with the increasing stresses imposed by fine spatial scales, the trees may be rapidly at risk from the microenvironment apart from climate change. However, to this day, there are only limited studies about how different leaf functional traits within adult-individual-level *P. chinensis* shift with microenvironmental gradients. The handful of functional traits studies of the *P. chinensis* from vertical gradients in China which measure water-use efficiency, leaf morphological structures and nutrient allocation strategies do suggest that there is a strong fierce pressure on plant function across vertical gradients (Deng et al., 2020). However, these studies usually focused on diverse size classes of trees with comparatively few leaf photosynthetic traits, which limit our capacity to understand intraspecific variations and niche differentiation in leaf functional traits. Therefore, there is a need to note that the investigation on the intraspecific variation and correlation between leaf functional traits will enable better understanding of the trade-off between adaptive characteristics and functional traits of *P. chinensis* for different vertical gradient.

Benefiting by using the canopy crane, we can access canopy leaves to measure leaf hydraulic and economics traits of sample trees of *P. chinensis* in the forest at different vertical heights directly (*in-situ* measurement). In the past, it was hard to measure photosynthesis-related traits *in-situ*, and gas exchange observations performed on branches that have been excised from the tree are widespread (*ex-situ* measurement) (Santiago and Mulkey, 2003; Missik et al., 2020), consequently. The method of cutting branches assumes that cutting branches underwater can minimize damage to the branches hydraulic system (Hanson et al., 2013; Sperry et al., 2016). However, some researchers suggest that compared with intact branches, leaf photosynthetic traits such as stomatal conductance and net rate of photosynthetic CO_2_ assimilation on detached branches are significantly distinct (Santiago and Mulkey, 2003; Missik et al., 2020). Related excision response could vary due to factors such as the length of the excised branches and the canopy position (Woodruff et al., 2004; Buckley, 2005; Missik et al., 2020). Regardless of the mechanisms, there is still less agreement about which species may be affected minimally this measurement. Thereby, it is reasonable to expect that large trees like the *P. chinensis* under greater risk of hydraulic failure may exhibits remarkably significant divergent results by using different measurement methods.

In this study, we assessed key leaf economics and hydraulic traits (**Table 1**) of adult trees of *P. chinensis* at three heights (**Table S1**) and addressed whether variation in leaf photosynthetic traits is determined by tree height, different measurements and their interaction. To achieve the above goals, we examined (i) the variations in leaf functional traits along vertical gradients at the individual species level; (ii) the interrelationships between different functional traits at the individual level; (iii) the effect of different measurement methods on photosynthetic physiological parameters of *P. chinensis*.

**Table 1:** Definitions of leaf traits used in this article together with abbreviations and units.

Leaf functional trait	Abbreviation	Unit	Functional significance
Leaf morphological and structural trait			
Leaf area	LA	cm^2^	Light interception and gas exchange in plants (Poorter et al., 2009)
Leaf mass per area	LMA	g·cm^-2^	A measure of investment in structure, supporting the carbon and nutrients within leaves (Wright et al., 2004)
Leaf Thickness	LT	cm	Water storage and photosynthetic efficiency (Wright et al., 2004)
Leaf tissue Density	LD	g·cm^-3^	Similar to LMA (Poorter et al., 2009)
Leaf Relative Water Content	LWC		Water storage and the ability to withstand drought (Wilson et al., 1999; Wang et al., 2022)
Stomatal Conductance	Gs	mmol·m^-2^s^-1^	Water-CO_2_ exchange (Brodribb and Holbrook, 2003)
Leaf photosynthetic physiological traits			
Maximal net assimilation rate per mass	*A*_mass_	nmol·m^-2^s^-1^	Carbon assimilation (Givnish, 1988)
Light saturation point	LSP	µmol·m^-2^s^-1^	Adaptation of plants to strong light (Givnish, 1988)
Light compensation point	LCP	µmol·m^-2^s^-1^	Tolerance of plant to low light levels (Givnish, 1988)
respiration rate per mass	Rd_mass_	nmol·m^-2^s^-1^	Tolerance of plant to low light levels (Givnish, 1988)
Leaf hydraulic traits			
Leaf osmotic potential	OP	MPa	Hydraulic safety of plants (Sack and Holbrook, 2006)
Midday leaf water potential	MWP	MPa	Hydraulic safety of plants (Sack and Holbrook, 2006)
Leaf chemical traits			
Mass-based leaf nitrogen content per mass	N_mass_	mg·g^-1^	Photosynthetic efficiency and nutrient status (Ripullone et al., 2003)
Photosynthetic nitrogen use efficiency	PNUE	μmol·m^-2^s^-1^	Carbon assimilation (Ripullone et al., 2003)

## Materials and methods

### Study site and design

We conducted this study in the Xishuangbanna National Natural Reserve (101°34’N, 21°36’E) in Yunnan Province, China, which is a virgin tropical seasonal rainforest and *P. chinensis* is the dominant canopy tree species therein (Tang et al., 2008; Deng et al., 2020). The Xishuangbanna National Natural Reserve experiences a tropical seasonal climate with an obvious dry season (November-April) and a rainy season (May-October) which provides 80% of annual precipitation; the mean annual temperature is 21°C, and the mean annual rainfall is 1532 mm (Deng et al., 2020).

The canopy crane is located in the same tropical seasonal rainforest (TCT7015-10E, Zoomlion Heavy Industry, Changsha, China), which maximum operational height is 80 m and the job length is 60 m. A dynamics plot of 1.44 ha was established in 2014, centered around the crane base, for plant species investigation. Among all these 6928 individuals of 217 woody tree species with a diameter at breast height (DBH) ≥ 1 cm in this plot, *P. chinensis* had the highest relative importance values and the largest basal area (Tang et al., 2008).

In July 2022, which is the main growing season for most local plants. We first selected 5 adult-individual *P. chinensis* from the plot and divided their canopy into three equal parts from top to bottom. Afterwards, we sampled and measured southfacing branches and sun-exposed leaves of the canopy of these 5 trees across three vertical gradients. Before measuring leaves when sunny days, we operated the crane and measured every selected tree height with a tapeline.

### Measurement of leaf photosynthetic physiological traits

After reaching the different canopy heights (**Table S1**) using the forest tower crane (TCT7015-10E, Zoomlion Heavy Industry, Changsha, China), we used both uncut and detached branches to inspect the influence of any height-related and method-related effect on the gas exchange parameters. The light-response curve was measured with a portable photosynthesis-fluorescence meter (LI-6800, LI-COR, Inc, Lincoln, NE, USA) and we maintained a stable CO_2_ concentration in the fluorescence leaf chamber (400-440ppm) with a CO_2_ buffer bottle. The relative humidity was 75%–90% (Deng et al., 2020; Shen et al., 2022), the air flow was 500 µmol·s^−1^, the leaf temperature was approximately 30°C and the VPD was 1.3 ± 0.2 kPa (Deng et al., 2020). After light adaptation under 1000 µmol·m^-2^s^-1^, the PPFD gradient was set as follows: 2300, 1800, 1300, 800, 600, 400, 200, 100, 50, 30, and 0 µmol·m^-2^s^-1^. Before measurement, the instrument needed to be matched. At each PPFD gradient, the leaves were allowed to balance for at least 3 minutes in view of the steadiness of stomatal conductance (Gs). We analyze the light response curves by using the model proposed by Ye (2007), as denoted as Ye model hereinafter, following measurement to calculate the maximal net assimilation rate (*A*_max_), the light saturation point (LSP), the light compensation point (LCP) and the respiration rate (Rd).

The maximal net assimilation rate per mass is calculated as follows:


$$A_{\text{mass}}=\frac{\text{the maximal net assimilation rate}}{\text{Leaf mass per area}}$$

### Measurement of leaf morphological and structural traits

The Gs was recorded when measuring the photosynthetic physiological traits of leaves. We took measurements of the leaf thickness (LT) using a digital micrometer (SWSIWI, China) and avoided the main leaf vein. Leaf lamina area (LA) was measured using a leaf area meter (LI-3000A, Li-Cor, USA). We also measured leaf relative water content by weighing fresh leaves, soaking them in ultra-pure water for 2 hours, weighing the saturated fresh weight, then drying at them 65°C for 72 hours and weighing the dry mass. From these measurements, we calculated leaf dry mass per area (LMA), leaf density (LD), and leaf relative water content (LWC).

The calculation equations are as follows:


$$\text{Specific leaf weight}\left(\text{LMA}\right)=\frac{\text{leaf dry mass}}{\text{leaf area}}$$

$$\text{Leaf density}\left(\text{LD}\right)=\frac{\text{leaf dry mass}}{\text{leaf area }\times \text{ leaf thickness}}$$

$$\text{Leaf relative water content}\left(\text{LWC}\right)=\frac{\text{leaf fresh mass}-\text{ leaf dry mass}}{\text{saturated leaf fresh mass}-\text{ leaf dry mass}}$$

### Measurement of leaf chemical traits

Nitrogen content (N_mass_) in the dried and ground leaves was analyzed using an elemental analyzer (Vario ISOTOPE Cube, Elementar Analysensysteme GmbH, Langenselbold, Germany).


$$\text{Photosynthetic nitrogen use efficiency}\left(\text{PNUE}\right)=\frac{\text{ maximal net assimilation rate per mass }}{\text{nitrogen content per unit leaf dry mass}}$$

### Measurement of leaf hydraulic traits

We measured midday leaf water potential (MWP) immediately after collecting leaves by using a pressure chamber (Model 1505, PMS, Albany, USA) between 12:00-1:30 PM. We also measured leaf osmotic potential (OP) by using a vapor pressure osmometer (Vapro 5600, Wescor, USA). For different canopy layers of each sampled tree, we measured three to five sun-exposed leaves. All measurements were taken on the same sunny day.

### Data analysis

Leaf trait data were tested for the normality of distribution using the Shapiro–Wilk tests. Initially multiple comparisons (ANOVA) of leaf traits at different heights were carried out using Tukey’s test with 95% confidence intervals. Then we conducted a principal component analysis (PCA) to examine how leaf traits varied among the different elevations. A permutational multivariate analysis of variance (PERMANOVA) was used to test the difference in leaf traits at different heights. To examine the relationships between different types of leaf functional traits at the individual level, we conducted Pearson correlation analysis. To investigate the impact of different measurement methods on the parameters of leaf photosynthetic-related traits, we employed a linear mixed model (Zuur et al., 2009), treating the individual as a random factor and canopy height and measurement method as fixed effects (Shi et al., 2021). We first constructed a model including all factors and their interactions, and then compared this with the original model after sequentially removing two-factor interactions and single factors, to test the significance of factors or interactions (Lu et al., 2020). Furthermore, we performed the same statistical analysis on the functional trait parameters obtained from detached measurements as from *in-situ* measurements, to test the differences in statistical results under different measurement methods. All data analysis and visualizations were carried out using R v.4.2.1 (R Core Team, 2022).

## Result

### Response of leaf functional traits to canopy height

**Figure 1:**
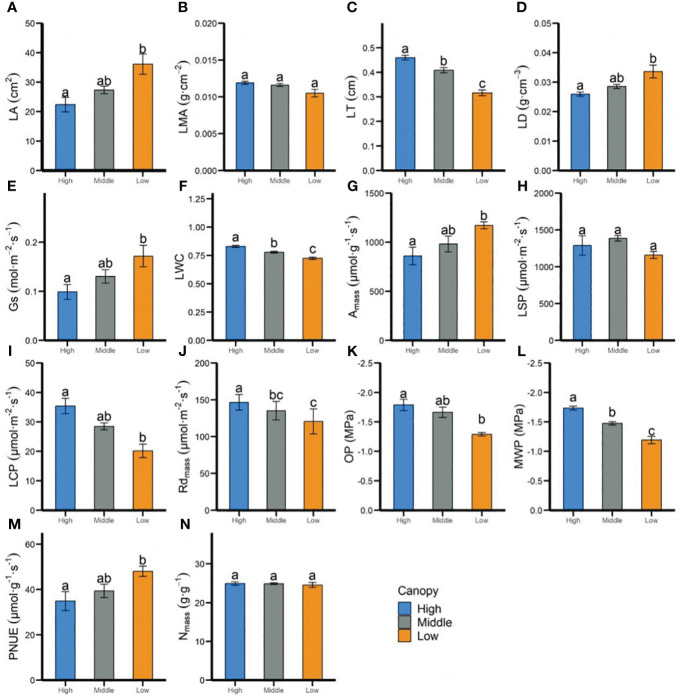
Differences in leaf functional traits of adult-individual level of *P. chinensis* at 3 vertical heights as measured *in-situ*. Differences for each trait were determined using the Tukey’s test, with a confidence level of 95%. High, high canopy; Middle, middle canopy; Low, lower canopy. LA, Leaf area **(A)**; LMA, leaf mass per area **(B)**; LT, leaf thickness **(C)**; LD, leaf density **(D)**; Gs, stomatal conductance **(E)**; LWC, leaf water content **(F)**; *A*_mass_, maximum net photosynthetic rate per unit mass **(G)**; LSP, light saturation point **(H)**; LCP, light compensation point **(I)**; Rd_mass_, dark respiration rate per unit mass **(J)**; OP, leaf osmotic potential **(K)**; MWP, midday leaf water potential **(L)**; N_mass_, nitrogen content per unit mass **(M)**; PNUE, photosynthetic nitrogen use efficiency **(N)**. Error bars represent standard errors. Significant differences between each height (ANOVA, *P*< 0.05) are indicated by different letters.

*In-situ* measurements revealed that most leaf functional traits of *P. chinensis* at the individual level varied significantly across different canopy heights. For instance, within the leaf morphological and structural traits, the LA, LD, and Gs increased 1.16-, 1.29-, and 1.73-fold, respectively (**Figures 1A–F**), from the low to high canopy, but the LT and LWC of high canopy were 1.45 and 1.13 times those at the lower canopy, oppositely. Leaf photosynthetic physiological traits, such as *A*_mass_, LCP, and Rd_mass_, showed significant difference between low and high canopy (**Figures 1G–J**), whereas no obvious discrepancies were observed in most of those parameters between the low and middle height (**Figures 1G–J**). Contrary to the trends for LWC, leaf hydraulic traits, including OP and MWP, declined with the increasing height (**Figures 1K, L**). Leaf chemical traits, such as N_mass_, was less affected by the vertical gradient.

### Correlation of leaf functional traits at three vertical heights

**Figure 2:**
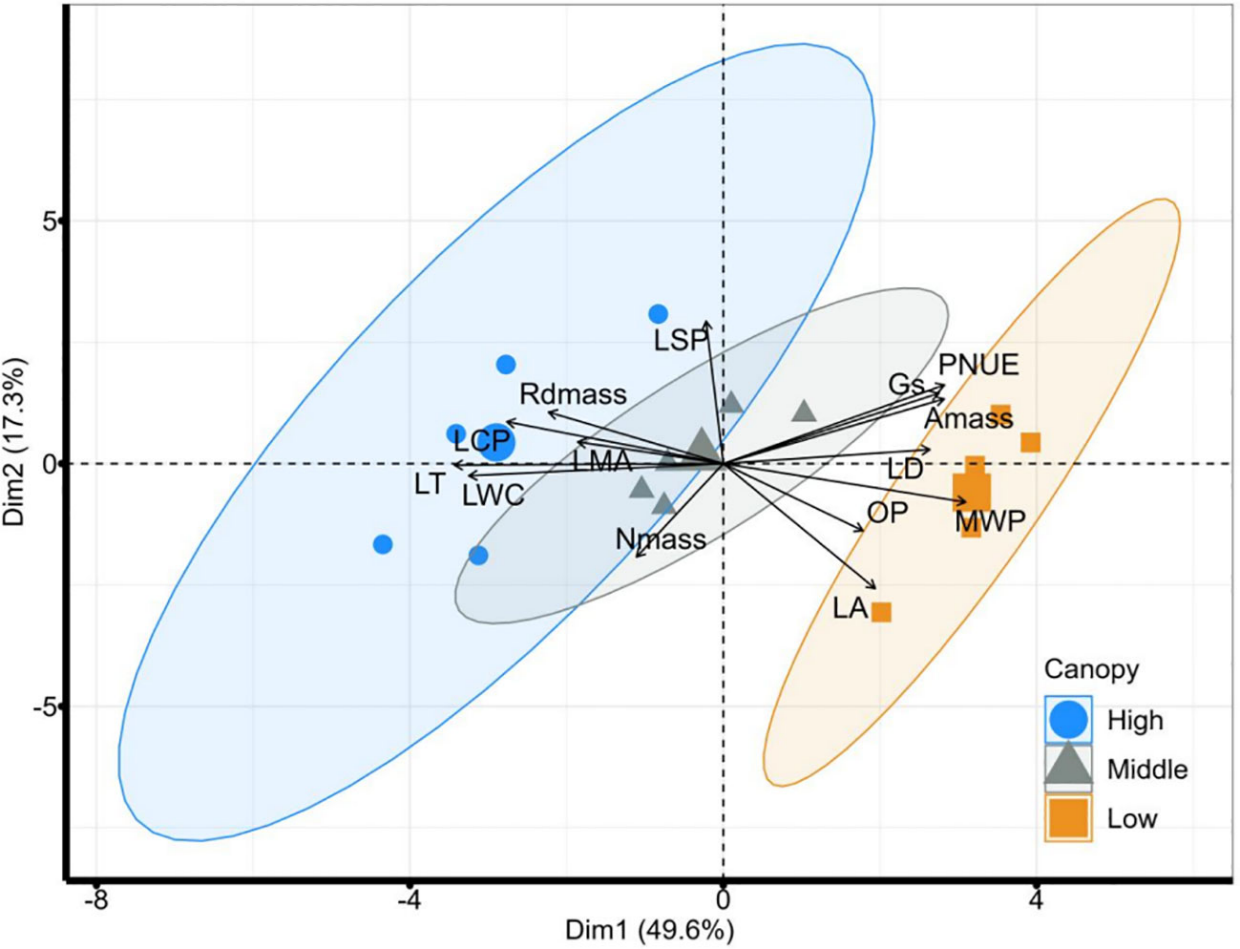
PCA analysis results of 14 leaf functional traits at different vertical heights measured *in-situ*. Blue, gray, and orange represent the high, middle, and lower vertical heights, respectively. The ellipses indicate a 95% confidence interval. High, high canopy; Middle, middle canopy; Low, lower canopy. LA, Leaf area; LMA, leaf mass per area; LT, leaf thickness; LD, leaf density; *A*_mass_, maximum net photosynthetic rate per unit mass; LSP, light saturation point; LCP, light compensation point; Rdmass, dark respiration rate per unit mass; Gs, stomatal conductance; LWC, leaf water content; MWP, midday leaf water potential; OP, leaf osmotic potential; N_mass_, nitrogen content per unit mass; PNUE, photosynthetic nitrogen use efficiency.

Our PCA revealed the individual-level variation of 14 leaf traits across multiple scales and divided them into two nearly decoupled dimensions (**Figure 2**). The first leading dimension of leaf adaptation to height, which accounted for 49.6% of the variation, was mainly dominated by leaf carbon assimilation and hydraulic safety (loaded traits such as Gs, PNUE, *A*_mass_, LD, MWP, OP, LSP, Rd_mass_, LCP, LMA, and LWC). The second principal component of resource acquisition, explaining 17.3% of the variation, was mainly represented by LA, LSP and N_mass_. The PCA revealed the existence of two leading independent dimensions of the variation in leaf traits. Along the first axis, the high height was separated from the other two heights, with significant differences among all three heights supported by PERMANOVA (*R* ^2 =^ 0.22, *P* = 0.036, **Table S2**).

**Figure 3:**
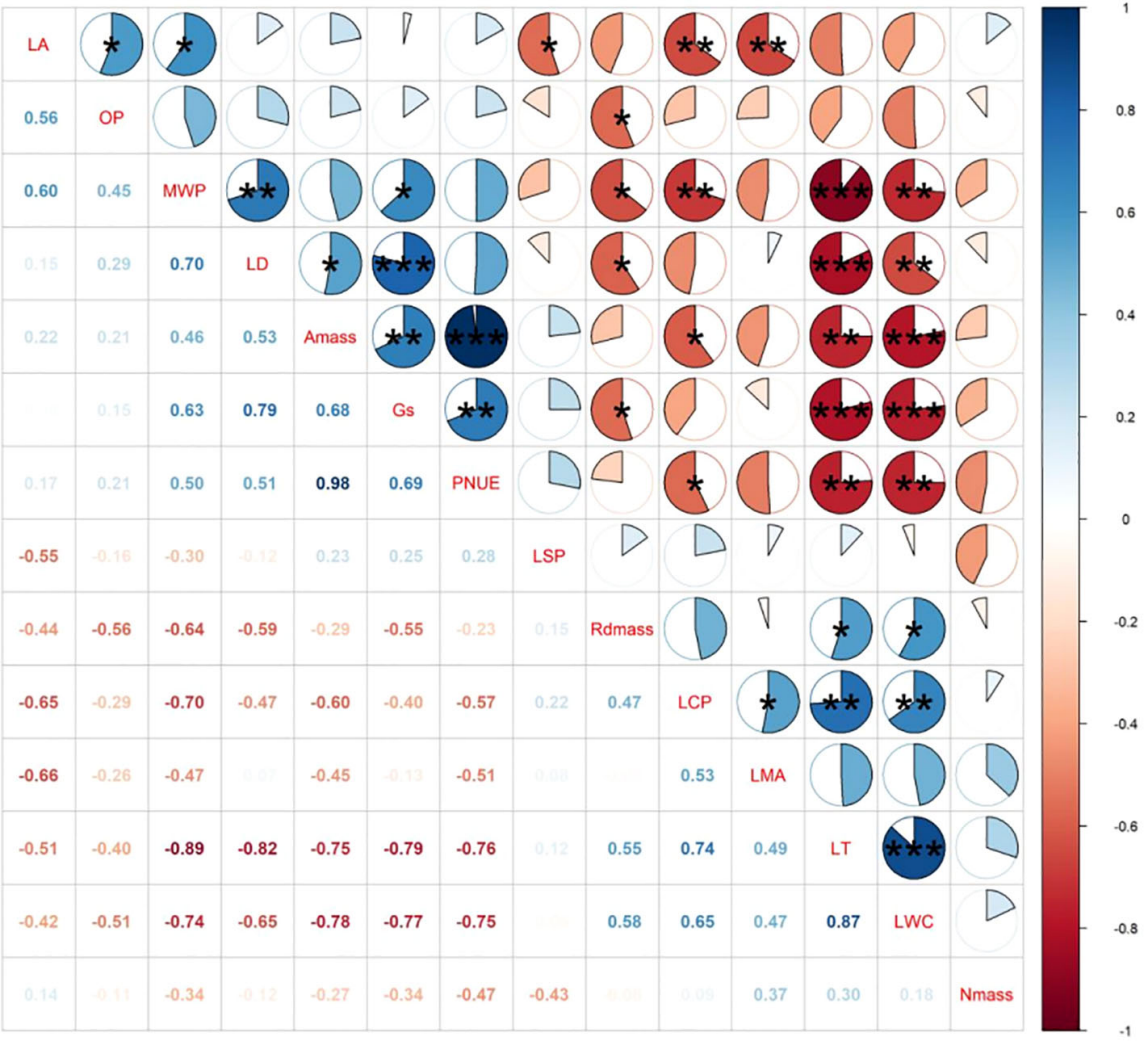
Results of the correlation tests between different leaf functional traits of *P. chinensis* measured *in-situ*. LA, Leaf area; LMA, leaf dry mass per area; LT, leaf thickness; LD, leaf density; *A*_mass_, maximum net photosynthetic rate per unit mass; LSP, light saturation point; LCP, light compensation point; Rd_mass_, dark respiration rate per unit mass; Gs, stomatal conductance; LWC, leaf water content; MWP, midday leaf water potential; OP, leaf osmotic potential; N_mass_, nitrogen content per unit mass; PNUE, photosynthetic nitrogen use efficiency. Significance is denoted by asterisks::*P< 0.05; **P< 0.01; and ***P< 0.001.

Among all leaf hydraulic traits, MWP had no correlation with OP (**Figures 2**, **3**). Among all leaf economic traits, LT was significantly negatively related to LD and Gs, whereas a positive relationship was found between *A*_mass_ and PNUE. Contrary to these significant correlations, neither MWP nor OP was uncorrelated with *A*_mass_.

### Leaf photosynthetic physiological parameters under different measurement methods

**Figure 4:**
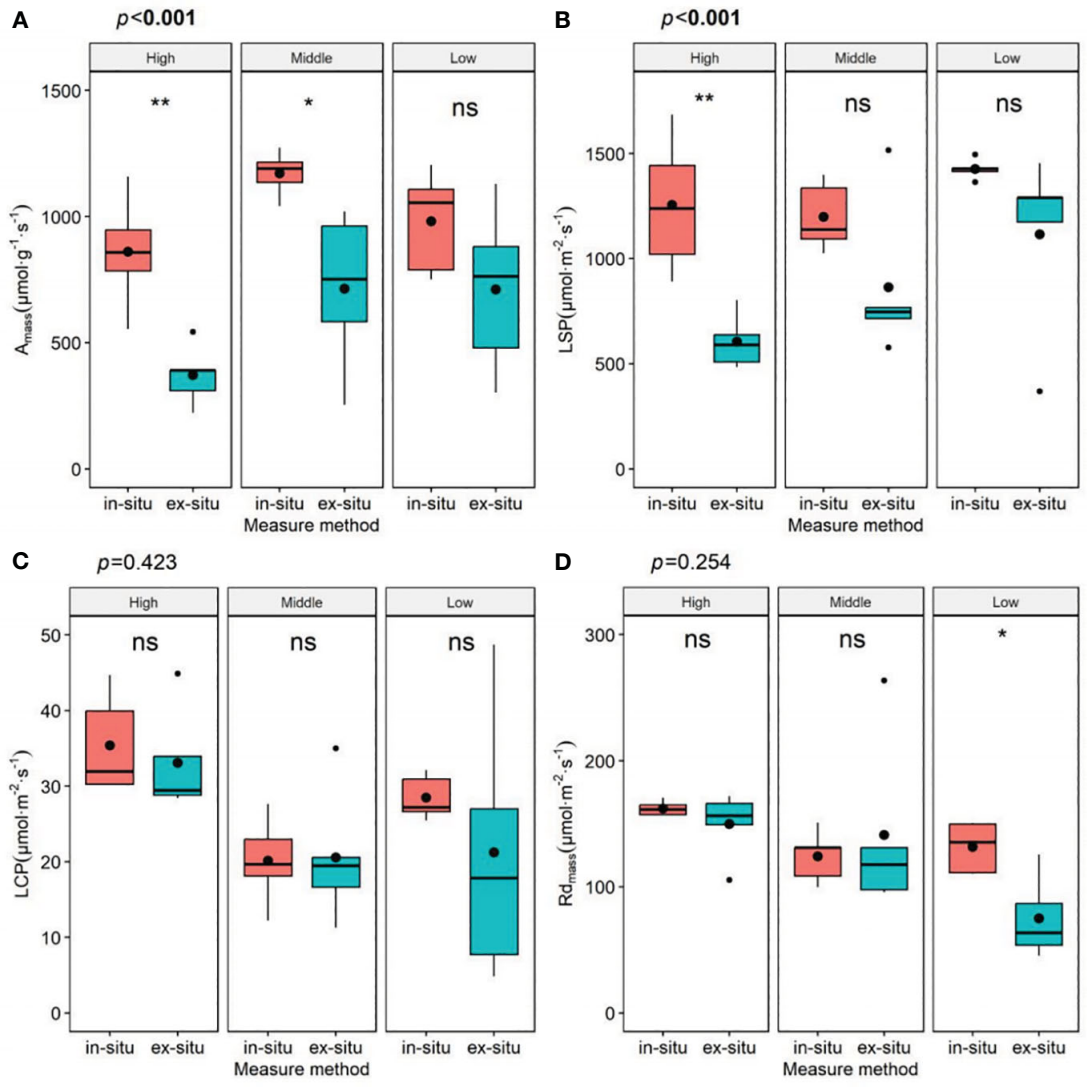
Parameters of light response curves of leaves at different heights under different measurement methods. *A*_mass_, maximum net photosynthetic rate per unit mass **(A)**; LSP, light saturation point **(B)**; LCP, light compensation point **(C)**; Rd_mass_, dark respiration rate per unit mass **(D)**; in-situ, *in-situ* measurement; ex-situ, *ex-situ* measurement; High: high canopy; Middle, middle canopy; Low, lower canopy. The P-value represents the impact of measurement methods on leaf functional traits of the overall tree canopy. The significance of different canopy layers are denoted by asterisks: *P< 0.05; **P< 0.01; ***P< 0.001; and ns, not significant.

There were significant differences in most parameters of the leaf photosynthetic physiological traits of *P. chinensis* between different measurement methods, particularly A_mass_ and LSP (**Figure 4**, **Table S4**). As shown in **Table S4**, from the lower to the upper canopy, the *ex-situ* measurements resulted in a decrease by 53.63%, 27.86%, and 38.05% in *A*_mass_, and a decrease by 50.00%, 19.21%, and 27.90% in LSP compared to the *in-situ* measurements, separately. We found that the multivariate permutation test of the differences in leaf functional traits at different canopy heights significantly changed when using detached branches (*R* ^2 =^ 0.15, *P*=0.12, **Figure S1**, **Table S2**), although different methods did not substantially alter the patterns of functional coordination between *A*_mass_ and leaf hydraulic traits (**Figure S2**).

## Discussion

### Changes in leaf functional traits with height

Leaf photosynthetic physiological traits such as LCP and Rd_mass_ are key indicators reflecting plant adaptation to light (Givnish, 1988). As canopy height increases, light availability also promoted. The transmittance of diffuse light varied from 0.3% to 100%, and the light intensity varied from 2.3 W·m^-2^ to 208W·m^-2^ (Deng et al., 2020; Shen et al., 2022). The leaves of *P. chinensis* exhibit low LCP and Rd_mass_ in the low canopy layer, which help to conserve carbon better than leaves in the high canopy and to gain carbon better at lower light levels (Koch et al., 2004). The trade-off between low LCP, respiration rates and high maximum photosynthetic rates of the leaves of *P. chinensis* in habitat shift from low canopy to high canopy reflected its change in ecological adaptation, which are similar to the pattern of the strategy of different sizes of trees from understory to canopy (Deng et al., 2020). The leaf morphological and structural trait is also likely to express the responses to the resource gradients (Wright et al., 2004; Reich, 2014), *P. chinensis* increased the thickness of the palisade tissue to enhance CO_2_ diffusion pathways and dissolution area (Zou et al., 2022), promoting leaf light capture ability and the concentration of photosynthesis-related enzymes (Jiao et al., 2022), leading to LT increased and LD declined (Poorter et al., 2009). In general, plants will invest more nitrogen for photosynthesis on strong light conditions to increase photosynthetic efficiency. However, in comparison with the high canopy, although the LSP and N_mass_ did not change with vertical gradients, *A*_mass_ and PNUE significantly decreased. These results are perhaps because the light conditions have reached at the status of saturation at the canopy layer, and the plants may allocate more nitrogen for non-metabolic functions such as light protection mechanisms (Roig-Oliver et al., 2021).

In addition to light condition, water resource along vertical gradient could shape the ecological adaption strategies (Koch et al., 2004; Pfautsch et al., 2018). Shen et al. (2022) have reported that the relative humidity above ground was obviously decreased as a result of increasing tree height in this plot, which ranged from 53.6% at 62m to 99.9% at 2 m. Hydraulic safety, as measured by leaf water potential like MWP and OP, is a trait linked to the degree of endurance to drought stress (Giles et al., 2021). We found that the leaves of *P. chinensis* in the low canopy adjust to being higher by decreasing leaf water potentials, resulting in lower MWP and OP in the high canopy (**Figure 1**). The unimodal pattern for leaf hydraulic traits is also consistent with the result from *Sequoia sempervirens* (Koch et al., 2004) and *Eucalyptus grandis* (Pfautsch et al., 2018). This indicates that large trees compensate for increasing water stress with height by adjusting their structures (Koch et al., 2004; Stovall et al., 2019). Moreover, leaves had a decrease in LA with tree height, but the LT still increased with tree height, suggesting that intraspecific variations, in addition to adaptations in light resources, might play an important role in adapting to maintain hydraulic safety function with increasing vertical gradients. Decreases in LMA with height were not found among different vertical layers, which suggests the decreases in MWP and OP with height were driven by adjustments to LT, LD and LA. This capacity may allow *P. chinensis* to have more flexibility in maintaining a trade-off between carbon balance and longevity, all of which change with LMA.

### Coordination of functional traits in *P. chinensis* at the individual species level

Leaf economics and hydraulic traits are important in plant growth and ecological adaptation. It has been proposed that they covary along a single axis of trait variation (Reich, 2014), which was based on the theory that hydraulic traits are the foundation of a key aspect of leaf economics spectrum (Wright et al., 2004). Although traits within each kind were closely associated, in this study, we found that leaf photosynthetic trait (*A*_mass_) and leaf hydraulic traits (MWP, OP) were statistically decoupled. This indicated that key leaf functional traits could not align themselves along a single axis of variation of *P. chinensis*, as recently proposed for functional trait variation across species (Reich, 2014). Instead, it suggests that different kinds of leaf functional trait can vary independently from each other even within an adult-individual level, which are consistent with studies that have observed independence among leaf functional traits across diverse sets of species and genotypes (Sack et al., 2003; Li et al., 2015; Blackman et al., 2016).

We found that leaf hydraulic traits such as OP were unrelated to net photosynthesis and stomatal conductance among *P. chinensis* individuals. These results support emerging reports of weak coordination between leaf hydraulic traits and leaf gas exchange across some sets of angiosperms (Gleason et al., 2016), which suggest that the driving factors linking hydraulic and gas exchange have not yet been fully understood. Yin et al. (2018) argued that adequate water availability may be a key factor leading to decoupling between functional traits by comparing and analyzing the relationship between the leaf economic and hydraulic traits in tropical-subtropical regions and the Loess Plateau. In moist area, plants tend to have more diverse and freer trait combinations to acclimate finely divided environmental gradients. For example, the *A*_mass_ may vary strongly with light availability (Feng et al., 2022), while the MWP changes with drought stress (Giles et al., 2021). We also found that *A*_mass_ was related to neither LMA nor N_mass_, indicating that photosynthetic capacity can vary among vertical gradients without covariation in key facets of total leaf investment. These results contrast previous research showed that positive relationships between leaf economics traits among species with structurally similar leaves (Wyka et al., 2012). At last, stomatal conductance was intimately related to LT and LD, implying that the leaf morphology and structure has a potential impact on the CO_2_-water exchange process (Zwieniecki and Boyce, 2014).

Among leaf functional traits comparisons across species, decoupling may be caused by the result of physical separation of leaf structures. Li et al. (2015) proposed that leaves typically contain two functional modules and represent two functional subsystems. The two may be influenced by evolutionary divergence. The upper palisade tissue is associated with leaf photosynthetic physiological traits, while the lower spongy tissue is associated with leaf hydraulic traits. Both of these may be influenced by evolutionary differences and the processes within each sublayer may not necessarily change simultaneously or at the same rate in different growth conditions. Such explanations are relatively limited within individual species, yet. Within species, independent functional trait dimensions may be caused by differential expression of genotypes, which is very attractive for understanding how multiple trait combinations are generated. Blackman et al. (2016) utilized this perspective to explain why the lack of significant correlation between vein density and maximum stomatal conductance across genotypes even if the functions on both sides of the leaf are the same. However, our results provide insight that the change in ecological adaption caused by microenvironment discrepancy on vertical gradients at individual scale may also alter the relationship between leaf structure, hydraulics, and gas exchange characteristics (Brodribb et al., 2013), which allows trees to adjust functions more freely to adapt to the particular set of fine-scale environment and thus maintain overall canopy performance and enhances ecological adaption in turn. It is apparent that these results are potentially extended to different large trees species, which have implications for understanding biogeochemical processes in global models.

### Impact of different measurement methods on the parameters of *P. chinensis*

Performing on detached branches is a common practice for gas-exchange measurements, especially for extremely tall trees like *P. chinensis*. These data allow us to further study the environmental adaptability and plasticity of plants (Missik et al., 2020), and provide a basis for local or even global terrestrial ecological models (Walker et al., 2014). However, the data indicate that the conclusions drawn from this method tend to cause significant biases (**Figure 4**, **Table S3**), which were also reported previously (Missik et al., 2020).

In **Table S3**, the data suggest that canopy position may also be the possible driver affecting measurement parameters. Species with high photosynthetic capacity also demonstrate high water-CO_2_ exchange capacity to avoid photosynthetic constraints (Xu et al., 2021). Large trees must transport water to higher heights to resist the effects of gravity and path length related resistance (Liu et al., 2019), thus more prone to hydraulic challenges such as xylem cavitation and embolism (Bennett et al., 2015; Liu et al., 2021). The leaves of the upper canopy of the *P. chinensis* have lower OP and MWP (**Figure 1**), indicating greater cavitation vulnerability. This to some extent explains why there is a greater deviation in the results *ex-situ* measured at the upper crown layer compared to the lower layer of the tree crown (**Figure 3**). Even though branches are immediately placed in water and recut after pruning from the tree, it may still cause xylem embolism and hydraulic conductivity (Santiago and Mulkey, 2003), which are closely related to Gs (Hernandez-Santana et al., 2016). Given that the Gs measured in *ex-situ* branches are greatly affected (**Table S4**), which in turn affects photosynthesis, it is not possible to accurately reflect the differences in leaf ecological adaptability of different canopy heights. Besides, a decrease in water transport capacity also affects the transport of hormones and nutrients (Santiago and Mulkey, 2003), thereby probably inhibiting photosynthesis (Nam et al., 2021).

Our results suggest that in trait-based ecological research, especially when it comes to leaf photosynthetic physiological traits, *in-situ* measurement should be used as much as possible. When on-site conditions are limited, we should cut branches that exceed the length of the vessel (Santiago and Mulkey, 2003). Theoretically longer detached branches could decrease biases, which is partly because that if the length of the excised branch is much longer than xylem elements, the leaves on the cut longer branches may be farther away from the xylem elements at the distal end of the branches, which may be less affected by embolism (Santiago and Mulkey, 2003; Missik et al., 2020). Unfortunately, cutting long branches is constrained by the current protection and management policies of *P. chinensis*. In addition, in light of the varying degrees of resection bias between species caused by differences in xylem (Hacke et al., 2001, Santiago and Mulkey, 2003; Missik et al., 2020), comparisons between species that rely on *ex-situ* branch data may be unreliable. For future research, we suggest conducting comparative tests on branches that can be used for *in-situ* measurement. Previous work has also shown that compared to observation results collected in the field shortly after branch cutting (Niinemets et al., 2005), pre-treatment of cut branches under low light and constant temperature for 2-3 days may reduce bias related to cutting, which is not allowed to observe the gas exchange rate at the *in-situ* stress level.

## Conclusion

Our study showed that *Parashorea chinensis* leaf functional traits varied substantially with different canopy heights in the same community. The individual variation could determine the photosynthetic capacity for *P. chinensis* to survive in environments with differential light and water availability and contributes to the evidence of ecological adaptation observed along those fine-scale environmental gradients. We also provided evidence that key leaf functional traits can vary independently even at the individual level, which may allow different resultant of adaptive traits in response to changing environments. In addition, the data also showed that different measurement results can significantly influence the results of the data analysis associated with measurement parameters, so differences in measurement methods should be weighed when determining the functional traits of *P. chinensis*. In summary, understanding individual variations in *P. chinensis* can contribute considerably to deeply understood ecological adaptation strategies of canopy species under the background of climate change.

## Data availability statement

The raw data supporting the conclusions of this article will be made available by the authors, without undue reservation.

## Author contributions

NJ: Conceptualization, Data curation, Formal analysis, Validation, Visualization, Writing – original draft, Writing – review & editing. XY: Investigation, Writing – original draft. JD: Investigation, Writing – original draft. MD: Conceptualization, Data curation, Investigation, Supervision, Visualization, Writing – original draft. YM: Writing – review & editing. LF: Writing – original draft. RB: Writing – original draft. JZ: Writing – original draft. JS: Writing – original draft. GD: Writing – original draft. HL:Conceptualization, Funding acquisition, Methodology, Supervision, Writing – original draft, Writing – review & editing.
