# Supplementary material for: Vertical variation in leaf functional traits of Parashorea chinensis with different canopy layers
Source: Front Plant Sci. 2024 Jan 29;15:1335524. doi: 10.3389/fpls.2024.1335524 (PMC10859428; doi:10.3389/fpls.2024.1335524)
Supplement: Supplementary file 1 [file DataSheet_1.docx]

**Supplementary material**

Jin et al., Vertical variation in leaf functional traits of *Parashorea chinensis* with different canopy layers.

**Tables**

**Table S1.** Vertical height (Mean values ± standard error; *n* = 5) of the *Parashorea chinensis* stand investigated in Xishuangbanna.

| Canopy | High | Middle | Low |
| --- | --- | --- | --- |
| Height (m) | 63.5±1.8 | 55.9±2.1 | 48.3±2.4 |

**Table S2.** The impact of different measurement methods on the results of the PERMANOVA analysis.

| Method | *in-situ* measurement | *ex-situ* measurement |
| --- | --- | --- |
| *R*^2^ | 0.22 | 0.15 |
| *P* | 0.036 | 0.12 |

**Table S3.** The impact of different measurement methods on the leaf functional traits parameters of the *Parashorea chinensis*. *A*_mass_: maximum net photosynthetic rate per unit mass; LSP: light saturation point; LCP: light compensation point; Rd_mass_: dark respiration rate per unit mass; Gs: stomatal conductance.

| Traits |  | Height | Method | Height × Method |
| --- | --- | --- | --- | --- |
| *A*_mass_ | *χ*2 | 16.374 | 23.647 | 1.704 |
|  | *P* | 0.0002 | 1.157e-06 | 0.427 |
| LSP | *χ*2 | 9.358 | 15.617 | 2.297 |
|  | *P* | 0.009 | 7.755e-05 | 0.317 |
| LCP | *χ*2 | 10.188 | 1.348 | 1.762 |
|  | *P* | 0.006 | 0.246 | 0.414 |
| Rd_mass_ | *χ*2 | 9.667 | 1.784 | 6.200 |
|  | *P* | 0.008 | 0.182 | 0.045 |

**Table S4.** Physiological traits at three canopy heights using different measurement methods. *A*_mass_: maximum net photosynthetic rate per unit mass; LSP: light saturation point; LCP: light compensation point; Rd_mass_: dark respiration rate per unit mass; Gs: stomatal conductance.

| Canopy | Method | *A*_mass_ | LSP | LCP | Rd_mass_ | Gs |
| --- | --- | --- | --- | --- | --- | --- |
| High | *In-situ* | 800.51±59.77 | 1208.31±120.69 | 35.40±2.63 | 146.51±10.68 | 0.105±0.02 |
| Middle | *In-situ* | 986.15±77.58 | 1426.31±18.41 | 30.91±2.93 | 144.26±16.11 | 0.13±0.01 |
| Low | *In-situ* | 1152.92±37.03 | 1198.52±64.54 | 20.12±2.30 | 120.64±16.93 | 0.17±0.02 |
| High | *Ex-situ* | 371.18±47.28 | 604.16±50.76 | 33.09±2.77 | 149.79±10.51 | 0.03±0.002 |
| Middle | *Ex-situ* | 711.36±130.61 | 1115.32±171.59 | 21.22±7.08 | 74.93±12.89 | 0.05±0.004 |
| Low | *Ex-situ* | 714.18±124.23 | 864.16±148.77 | 20.58±3.53 | 141.09±27.98 | 0.06±0.007 |

**Figures**


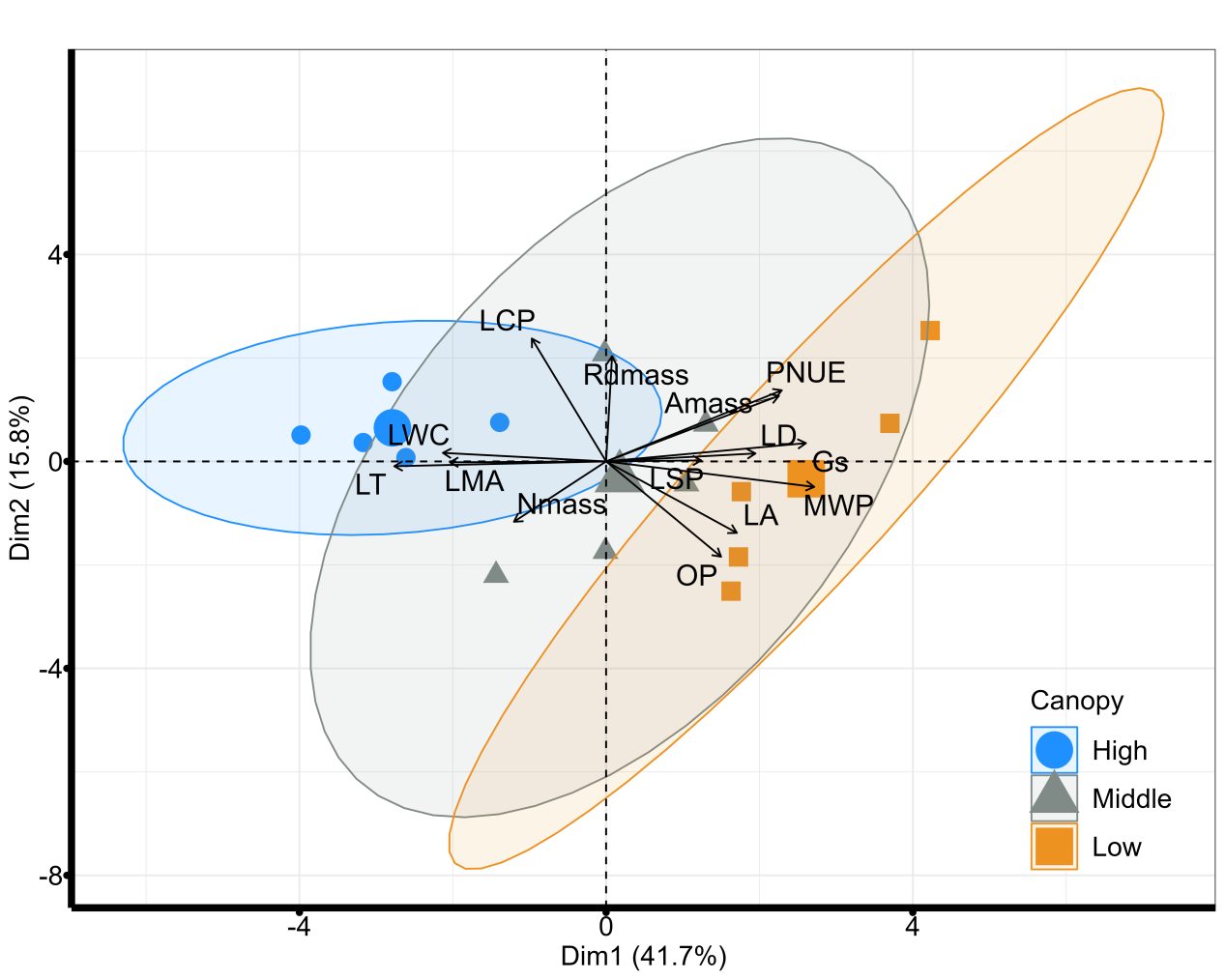


**Figure S1.** PCA analysis results of 14 leaf functional traits at different canopy heights under *ex-situ* measurement.

Blue, gray, and orange represent the high, middle, and lower vertical heights, respectively. The ellipses indicate a 95% confidence interval. L: lower canopy; M: middle canopy; H: high canopy. LA: Leaf area; LMA: leaf dry mass per area; LT: leaf thickness; LD: leaf density; *A*_mass_: maximum net photosynthetic rate per unit mass; LSP: light saturation point; LCP: light compensation point; Rd_mass_: dark respiration rate per unit mass; Gs: stomatal conductance; LWC: leaf water content; MWP: midday leaf water potential; OP: leaf osmotic potential; N_mass_: nitrogen content per unit mass; PNUE: photosynthetic nitrogen use efficiency.


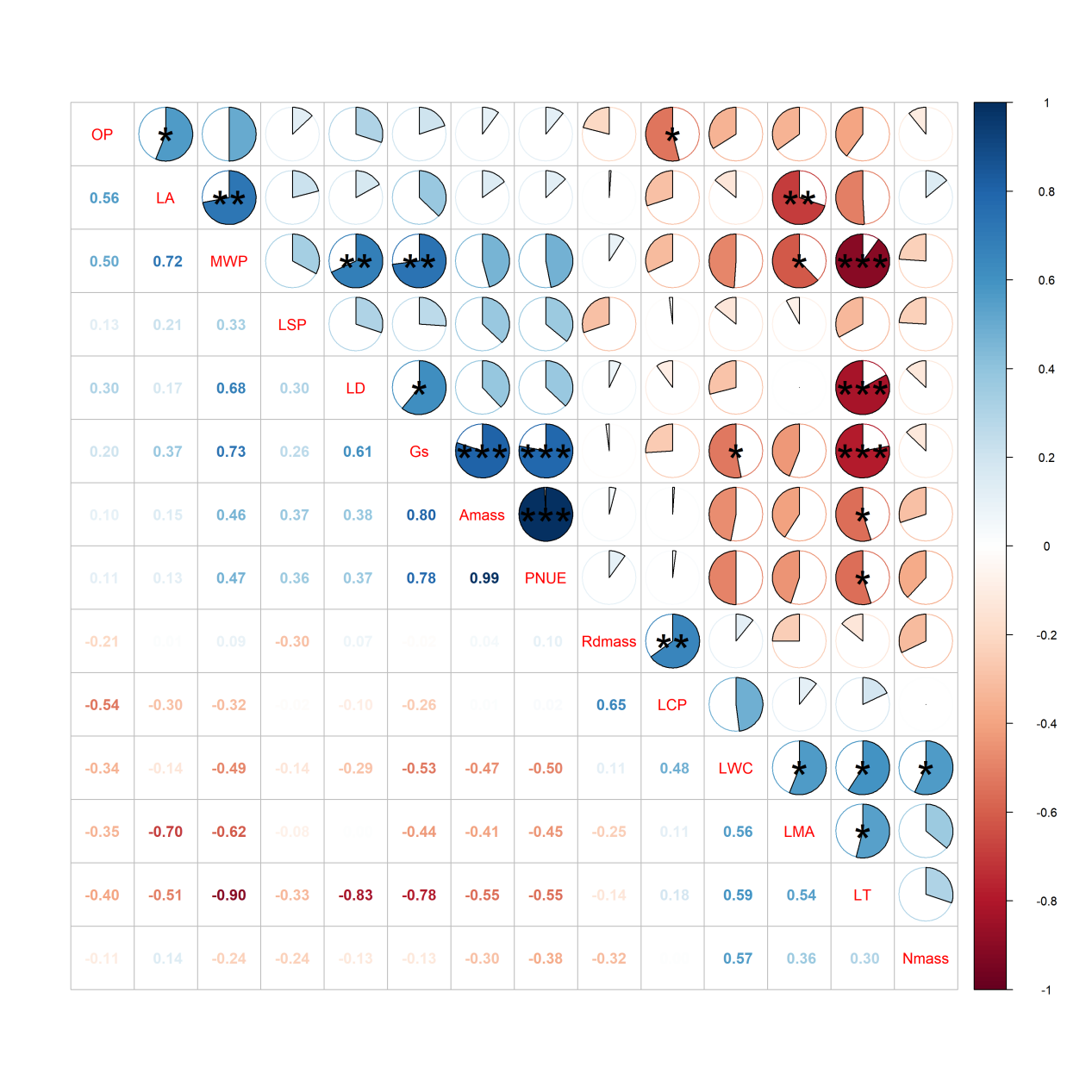


**Figure S2.** Results of the correlation analysis among various leaf functional traits in *Parashorea chinensis*.

LA: Leaf area; LMA: leaf mass per area; LT: leaf thickness; LD: leaf density; *A*_mass_: maximum net photosynthetic rate per unit mass; LSP: light saturation point; LCP: light compensation point; Rd_mass_: dark respiration rate per unit mass; Gs: stomatal conductance; LWC: leaf water content; MWP: midday leaf water potential; OP: leaf osmotic potential; N_mass_: nitrogen content per unit mass; PNUE: photosynthetic nitrogen use efficiency. Significance is denoted by asterisks: **P* < 0.05; ***P* <0.01;and ****P* < 0.001.
